# Acupuncture for cancer patients with nausea and vomiting: A protocol for systematic review and meta-analysis

**DOI:** 10.1097/MD.0000000000031478

**Published:** 2023-01-20

**Authors:** Sung-A Kim, Sabina Lim, Tiana Won, Sujung Yeo

**Affiliations:** a Department of Clinical Korean Medicine, Graduate School, Kyung Hee University, Dongdaemun-gu, Seoul, South Korea; b Department of Meridian and Acupoint, College of Korean Medicine, Kyung Hee University, Seoul, Republic of Korea; c Research Group of Pain and Neuroscience, WHO Collaborating Center for Traditional Medicine, East-West Medical Research Institute, Kyung Hee University, Seoul, Republic of Korea; d Department of Meridian and Acupoint, College of Korean Medicine, Sangji University, Wonju, Republic of Korea.

**Keywords:** acupuncture, cancer, meta-analysis, nausea, systematic review, vomiting

## Abstract

Nausea and vomiting are among the most common adverse effects experienced by cancer patients undergoing treatment worldwide. Their treatment with pharmacologic therapy can often be complicated by medication interactions and other unwanted side effects. The aim of this systematic review and meta-analysis protocol is to assess the effectiveness and safety of acupuncture therapy for treating nausea and vomiting in patients with cancer.

**Methods:**

Three electronic databases and 2 clinical registry platforms will be searched from inception to May 2022: the MEDLINE via PubMed, Embase via Ovid, the Cochrane Central Register of Controlled Trials via the Cochrane Library, the World Health Organisation International Clinical Trials Registry Platform, and National Institutes of Health Clinical trials.gov. Search terms will include nausea, vomiting, cancer, and acupuncture. Two researchers will independently select studies, extract data and assess the risk of bias. The primary outcome will be the incidence of nausea and/or vomiting or other validated outcome measures. Meta-analysis will be carried out using RevMan V.5.4. The quality of evidence from randomized clinical trials will be evaluated with the Grading of Recommendations Assessment, Development and Evaluation System tool.

**Results:**

The results will provide a high-quality synthesis of evidence for clinicians in the field of oncology.

**Conclusion:**

The conclusion is expected to provide evidence to determine whether acupuncture is an effective and safe treatment for cancer patients with nausea and vomiting.

## 1. Introduction

Nausea and vomiting are afflicting symptoms experienced by >80% of cancer patients.^[1]^ They are induced not only by emetogenic antitumor treatment such as chemotherapy, radiotherapy, and surgery but by cancer itself, independent of the treatment agent. These symptoms can lead to poor compliance with anticancer treatment as they result in metabolic imbalance, malnutrition, dehydration, and an immunodeficient state.^[2]^ 5-hydroxytryptamine type 3 receptor antagonists and neurokinin-1 receptor antagonists recommended as antiemetic prophylaxis in National Comprehensive Cancer Network guidelines have comparatively reduced emesis but not nausea.^[3]^ Moreover, antiemetic agents can produce side effects including electrocardiography change, sedation, headache, and constipation.^[4,5]^

Acupuncture has become an alternative non-pharmacological therapy to improve nausea and vomiting for cancer patients. The anti-emetic effects of acupuncture apparently stem from the resultant increase in hypophysial secretion of beta-endorphins and ACTH, with subsequent inhibition of the chemoreceptor trigger zone and vomiting center.^[6]^ Considering the autonomic nervous system is involved in the peripheral mechanism of nausea and vomiting, vagal modulation through Neiguan (PC6) has been proved effective.^[7]^ Furthermore, acupuncture has fewer side effects than antiemetics, which may be the most advantageous factor compared with medication for cancer patients.^[8]^

Previous systematic reviews were conducted to establish the effectiveness and safety of acupuncture on nausea and vomiting of cancer patients, however, they did not comprehensively cover the various emetogenic aspects of cancer and it have never been performed with a meta-analysis to our knowledge. More rigorous randomized clinical trials (RCTs) published recently have not been included in previous systematic reviews. For example, a RCT published in 2020 identified that cancer patients treated with acupuncture experienced significantly less severe chemotherapy-induced nausea and vomiting and it suggested at least 3 weeks of follow-up would be needed to confirm the significance of clinical effect.^[9]^

Therefore, we designed this study to determine the efficacy and safety of acupuncture treatment in the management of cancer patients with nausea and vomiting based on the most recent evidence and to investigate the most effective acupuncture points and treatment regimens to suggest more practical therapy for clinical care.

## 2. Methods and analysis

### 2.1. Study registration

The protocol for this systematic review and meta-analysis has followed guidelines of the Preferred Reporting Items for Systematic Reviews and Meta-Analysis (PRISMA) protocols.^[10]^ The review will be performed in line with the PRISMA declaration guidelines.^[11]^ We also registered this protocol in the International Prospective Register of Systematic Review 2022 with the registration number CRD42022333811.

### 2.2. Inclusion criteria for study selection

#### 2.2.1. Type of study.

Only RCTs of acupuncture treatment for cancer patients with nausea and vomiting without restrictions on publication language will be eligible for inclusion.

#### 2.2.2. Type of participant.

Patients with all types of cancer complaining of nausea and vomiting will be enrolled, regardless of age, gender, nationality, ethnicity, and etiology.

#### 2.2.3. Type of intervention.

We defined acupuncture treatment as those therapies involving the insertion of needles, including manual acupuncture, electroacupuncture, auricular acupuncture and laser acupuncture. Other therapy that doesn’t puncture the skin or combines other stimulation methods, including acupressure, transcutaneous electrical acupoint stimulation, warm needling, plum blossom needle, fire needling, cupping and moxibustion, will be excluded. Comparison interventions, including active therapies, sham acupuncture (pseudo-acupuncture interventions, non-penetrating sham acupuncture, sham acupuncture at non-acupoints, sham acupuncture at selected acupoints, and needling at inappropriate acupoints), and no treatment, will be included.^[12]^ Acupuncture either used alone or combined with other therapies which are the same as the comparison intervention will also be included.

#### 2.2.4. Type of outcome measure.

Incidence of nausea and/or vomiting or other validated outcome measures will be the primary outcomes. Characteristics indicating the severity of nausea and/or vomiting, such as duration, frequency, stage or intensity of symptoms will be included as secondary outcomes.

### 2.3. Search methods for identification of studies

#### 2.3.1. Electronic data sources.

Studies published up to May 1, 2022 will be retrieved from the following 3 databases: MEDLINE via PubMed, Embase via Ovid, and the Cochrane Central Register of Controlled Trials. Two clinical registries including National Institutes of Health clinical registry (Clinical trials.gov) and The World Health Organisation International Clinical Trials Registry Platform will also be screened to reduce the risk of publication bias.

#### 2.3.2. Searching other resources.

Potentially missing eligible studies will be manually scanned as well from the reference lists of other systematic reviews and the relevant conference proceedings.

### 2.4. Search strategy

The following searching terms will be used: cancer, tumor/tumor, neoplasm, nausea, vomiting, emesis, acupuncture, randomized controlled trial, etc. These terms will be used alone or in combinations, using “and,” “or.”

### 2.5. Data collection and analysis

#### 2.5.1. Selection of studies.

**Figure 1.**
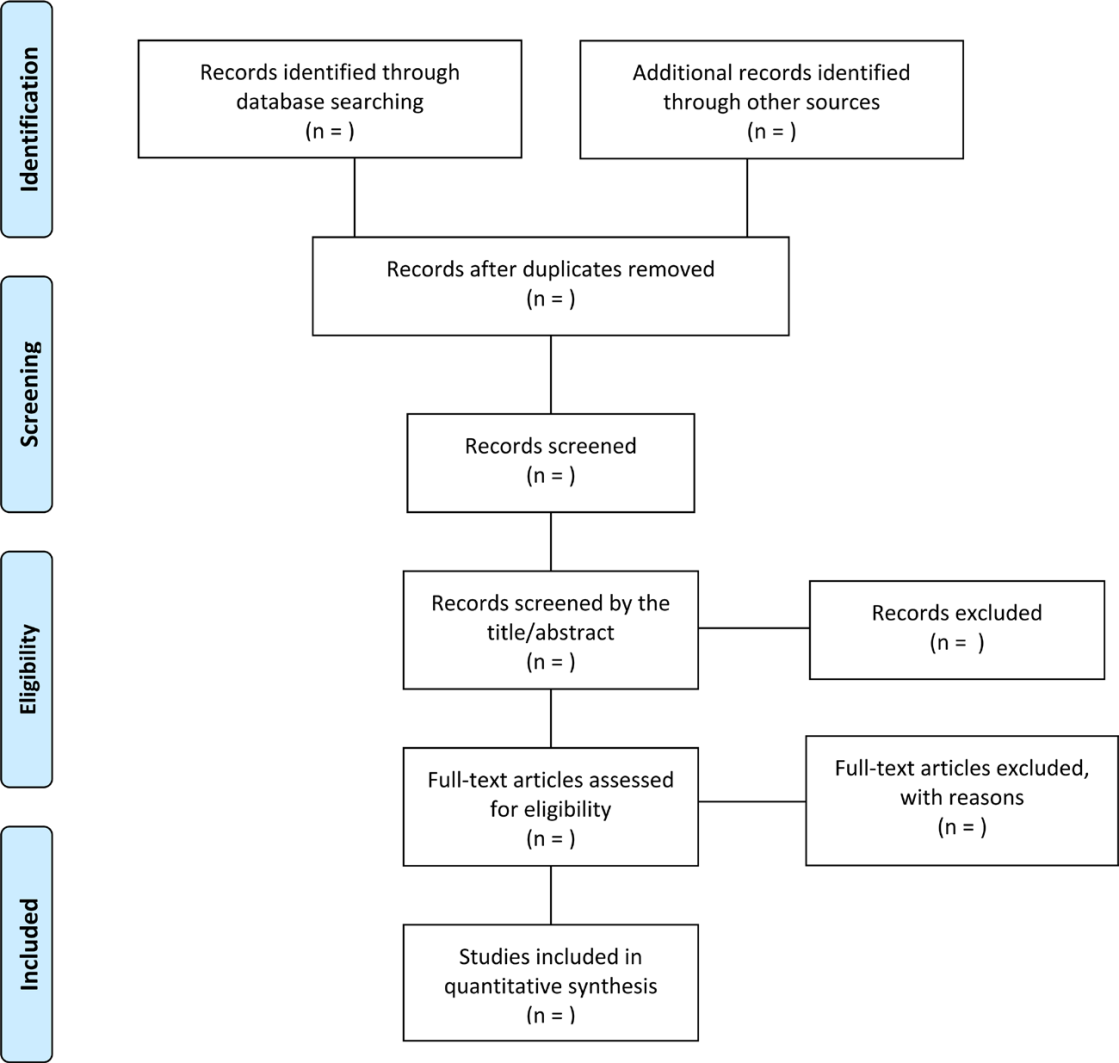
Flow chart of the trial selection process.

The titles and abstracts of studies will be browsed by 2 independent researchers (SAK and SBNL), after retrieving all eligible studies in Endnote 20 (Clarivate Analytics) and eliminating duplicate publications. Full texts of all potentially eligible studies will be scanned. Any disagreement on data selection will be solved with the arbiter (YSJ). A PRISMA flow diagram will show the study search and screening process (Fig. 1).

#### 2.5.2. Data extraction and management.

Data will be extracted by 2 researchers (SAK and SBNL) following the form: author, year of publication, country, participant characteristics, sample size, randomization, cancer type, emetogenic factor, interventions, control, outcome measures and other methodological information. A third reviewer (YSJ) will judge the disagreements. The analysis and synthesis will be conducted with RevMan V.5.4 software (Cochrane Collaboration, Oxford, UK) after transferring the data.

#### 2.5.3. Assessment of risk of bias in included studies.

Two authors (SAK and SBNL) will independently evaluate the risk of bias using the Cochrane Collaboration’s tool assessment method.^[13]^ The following biases will be assessed: selection bias, performance bias, attrition bias, detection bias, reporting bias, and other sources of bias. The judgement will include low risk, high risk, and unclear. The arbiter (YSJ) will solve any disagreement on assessment of bias.

#### 2.5.4. Measures of treatment effect.

Continuous data will be presented as standard mean difference 95% confidence intervals, while dichotomous data will be measured as odds ratio or risk ratio and 95% confidence interval.

#### 2.5.5. Unit of analysis issues.

Each individual participant will be the unit of analysis.

#### 2.5.6. Assessment of heterogeneity.

We will appraise heterogeneity for quantifying inconsistency by the *I*^2^ test and detect statistical heterogeneity with standard *x*^2^ test. If *I*^2^ value >50% or the *P* value is <.1, studies will be regarded to have significant heterogeneity and subgroup analysis will be conducted to analyze the potential causes. When *I*^2^ value <50% or the *P* value exceeds .1, studies will be considered to have homogeneity and the fixed-effects model will be applied.

#### 2.5.7. Assessment of reporting biases.

If >10 studies are included, the reporting biases will be assessed with funnel plots.

### 2.6. Data synthesis

RevMan V.5.4 will be used to implement the data synthesis for a meta-analysis. If substantial statistical heterogeneity is found, the random-effects model will be used, otherwise, the fixed-effects model will be used. Descriptive analysis will be carried out if the data between the trials are inappropriate to be merged.

### 2.7. Subgroup and sensitivity analysis

Subgroup analysis will be performed if the extracted data are sufficient, according to the different outcomes or control interventions. Sensitivity analyses will be conducted to evaluate the study design, impact of sample size, and methodological quality, and to identify the robustness of the data synthesis if possible.

### 2.8. Grading the quality of evidence

The Grade of Recommendations Assessment, Development and Evaluation will be the tool for evaluating the quality of the evidence.^[14]^ The assessments will be categorized into 4 levels (very low, low, moderate, or high) on the following domains: study design limitation, results inconsistency, indirectness, imprecision, and publication bias.

### 2.9. Ethics and dissemination

Since the patient data cannot be individualized, ethical approval is not required. The essential protocol amendments will be documented in the full review. The results will be disseminated in a peer-reviewed journal.

## 3. Discussion

This study will evaluate the effectiveness and safety of acupuncture treatment for cancer patients with nausea and vomiting. Existing reviews have mainly suggested the effect of Neiguan (PC6), but other various treatments are often currently used in clinical practice such as electroacupuncture or other acupuncture points.^[15]^ This review will try to provide information on the various acupuncture points and acupuncture types applicable to the treatment of nausea and vomiting, not just the effects of acupuncture on nausea and vomiting symptoms. This research could have practical and valuable implications for the use of acupuncture in the field of oncology, which will help inform the use of acupuncture as an alternative therapy for nausea and vomiting in cancer patients and potentially provide more options for symptom relief.

## Author contributions

**Conceptualization:** Sung-A Kim, Sujung Yeo.

**Data curation:** Sabina Lim, Sung-A Kim.

**Formal analysis:** Sabina Lim.

**Investigation:** Sabina Lim.

**Methodology:** Sabina Lim, Sung-A Kim, Tiana Won.

**Resources:** Sujung Yeo.

**Supervision:** Sabina Lim, Sujung Yeo.

**Validation:** Tiana Won.

**Visualization:** Sung-A Kim

**Writing – original draft:** Sabina Lim, Sung-A Kim.

**Writing – review & editing:** Sabina Lim, Tiana Won, Sujung Yeo.
